# Current understanding of the role of the cell wall in *Cuscuta* parasitism

**DOI:** 10.1111/plb.70059

**Published:** 2025-06-04

**Authors:** M. Takagawa, R. Yokoyama

**Affiliations:** ^1^ Faculty of Science Tohoku University Sendai Japan; ^2^ Graduate School of Life Sciences Tohoku University Sendai Japan

**Keywords:** *Cuscuta*, haustoria, parasite, plant cell wall

## Abstract

The plant cell wall (CW) plays a crucial role in many aspects of parasitism by the obligate stem parasite, *Cuscuta*. *Cuscuta* parasitism begins with tight coiling around the host stem by the strong tensile force of the thickened inner cell wall layers, and attachment to the host surface using secretory CW components. Subsequently, invasion of a feeding structure called the haustorium is facilitated by degradation and modification of host CWs. Furthermore, haustoria‐derived search hyphae transdifferentiate into tracheary elements of the secondary cell walls (SCW). SCW provides mechanical strength and hydrophobicity to the tracheary element. Therefore, *Cuscuta* can draw fluids from the host through the tracheary element. Thus, the parasitic processes in *Cuscuta* are closely linked to their CW structures and functions. In this review, we comprehensively summarise the role of the CW in each parasitic process of *Cuscuta* and provide details on the current understanding of *Cuscuta* parasitism.

## Graphical abstract

We comprehensively summarise the roles of plant cell wall in key processes of parasitism by the obligate stem parasite *Cuscuta*.

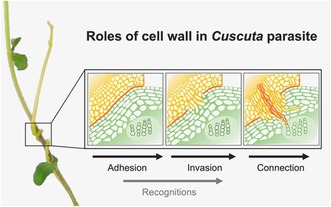

## INTRODUCTION

Plant cell walls (CWs) are central to maintaining and changing plant cell shape (Cosgrove 2022, 2024). The framework of the CW is mainly composed of cellulose microfibrils and matrix polysaccharides such as hemicelluloses and pectins, which form a three‐dimensional interwoven network structure (Delmer *et al*. 2024). The network structure resists turgor‐generated tensile forces in the plane of the wall while allowing relaxation for cell expansion and changes in cell shape. The CW is formed as a more functional network structure by the addition of a variety of polysaccharides, highly glycosylated proteins, and phenolic compounds. Furthermore, the composition of the CW is differentially regulated depending on cell type and different growth and development stages (Dauphin *et al*. 2022). Some types of cells, such as xylem cells, produce secondary cell walls (SCWs) once the cells stop expanding. SCWs are composed of an abundance of cellulose, xylan, and glucomannan and are embedded with lignin to strengthen the cell wall structure (Kumar *et al*. 2016; Meents *et al*. 2018; Zhong *et al*. 2019; McFarlane 2023). Lignified SCWs provide plant cells with mechanical support and the ability to transport solutes.

Cell properties determined by the CW play a key role in several aspects of plant growth, cell differentiation, intercellular communication, and adaptation to a changing environment. Plant cells are functionally shaped and enlarged during development by finely tuned cell‐wall deformation patterns (Cosgrove 2024). Additionally, plants can perceive the external environment by monitoring the state of their CW and adjusting their composition and structure in response to the environment (Voxeur & Höfte 2016; Vaahtera *et al*. 2019; Du *et al*. 2022). The CWs of host plants are degraded by various enzymes secreted by pathogens, such as fungi, bacteria and nematodes. The host plant recognises the oligosaccharides released by the degradation of CWs as infection signals and strengthens CWs against pathogen infection through callose and lignin deposition or cross‐linking of structural proteins (Vorwerk *et al*. 2004; Bellincampi *et al*. 2014; Voigt 2014; Hamann 2012).

Plant cell wall play a crucial role in plant parasitism, particularly in the interaction mechanisms between parasitic plants and hosts (Hartenstein *et al*. 2023). Parasitism is a highly successful life strategy among land plants, and approximately 1% of angiosperm species are parasitic, deriving all or part of their water and nutrients from host plants (Westwood *et al*. 2010). Among them, *Cuscuta* species are obligate plant‐stem parasites, characterised by thin stems, almost no roots and leaves. The genus *Cuscuta* comprises approximately 200 species with a worldwide distribution, some of which subsist on a wide range of host plant species and can cause significant economic and ecological losses (Dawson *et al*. 1994; Kaiser *et al*. 2015). Understanding the molecular basis of parasitism by *Cuscuta* species is of critical for the crop production and environmental preservation viewpoint.

**Fig. 1:**
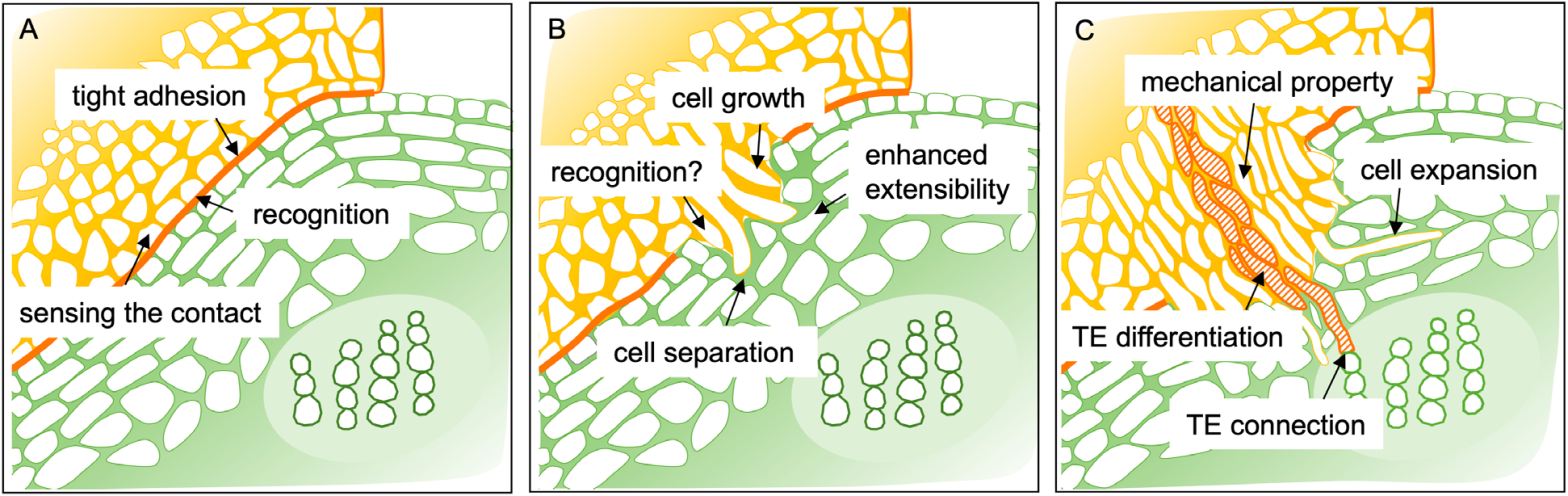
Parasitic processes involved in CW functions of *C. campestris*. (A) Adhesion and recognition phase; (B) Haustorial invasion phase; (C) TE differentiation and connection phase. Yellow and green areas show *Cuscuta* and host tissue, respectively.

*Cuscuta* species parasitise the host plant using an invasive organ known as the haustorium (Balios *et al*. 2025). After the haustoria penetrates into the host tissue, the search hyphae emerging from the haustoria elongate and spread within the host tissue. Upon reaching the host vasculature, the search hyphae differentiate into xylem and phloem hyphae, establishing connections with the host's vascular system. Previously, we reported that haustoria invasion into host tissues is facilitated by the degradation and modification of host CWs in *Cuscuta campestris* (Yokoyama *et al*. 2021, 2022). We also performed transcriptomic analysis using an *in vitro* induction system in the haustorium of *C. campestris* and characterised the cell wall‐related genes involved in the differentiation of xylem hyphae, which directly absorb water and minerals from xylem vessels of the host (Kaga *et al*. 2020). In addition to the xylem‐mediated transport of water and minerals, various phloem‐mobile compounds translocate from the host to the parasite through symplastic connections via plasmodesmata (Shimizu & Aoki 2019). Although not identified, the formation of interspecific plasmodesmata is also essential for the unique gene sets involved in cell wall metabolism at the site of contact (Fischer *et al*. 2021). As described above, CW plays a significant role in various aspects of parasitism in *Cuscuta* species, which are obligate plant‐stem parasites (Dawson *et al*. 1994). We focus on four major parasitic processes (adhesion, recognition, invasion, and connection) in *Cuscuta* spp. (Fig. 1). The adhesive process is involved in the tight coiling of the *Cuscuta* stem around the host stem and the formation of *Cuscuta* adhesive tissues, characterised by the presence of secretory cells and the elongation of cells in the epidermal and cortical layers (Shimizu & Aoki 2019). *Cuscuta* species and host plants are thought to recognise each other when in direct surface contact or immediately after the penetration of haustoria into host tissues, defined as the process of recognition. In the invasion process, haustoria invade the host and search hyphae elongate within the tissues, while in the connection process, search hyphae differentiate into vascular hyphae, followed by the establishment of a vascular connection between the parasite and the host plant. In this review, we show how each parasitic process of *Cuscuta* is tightly linked to CW structures and functions.

## ADHESION BETWEEN *CUSCUTA* AND THE HOST PLANT

**Fig. 2:**
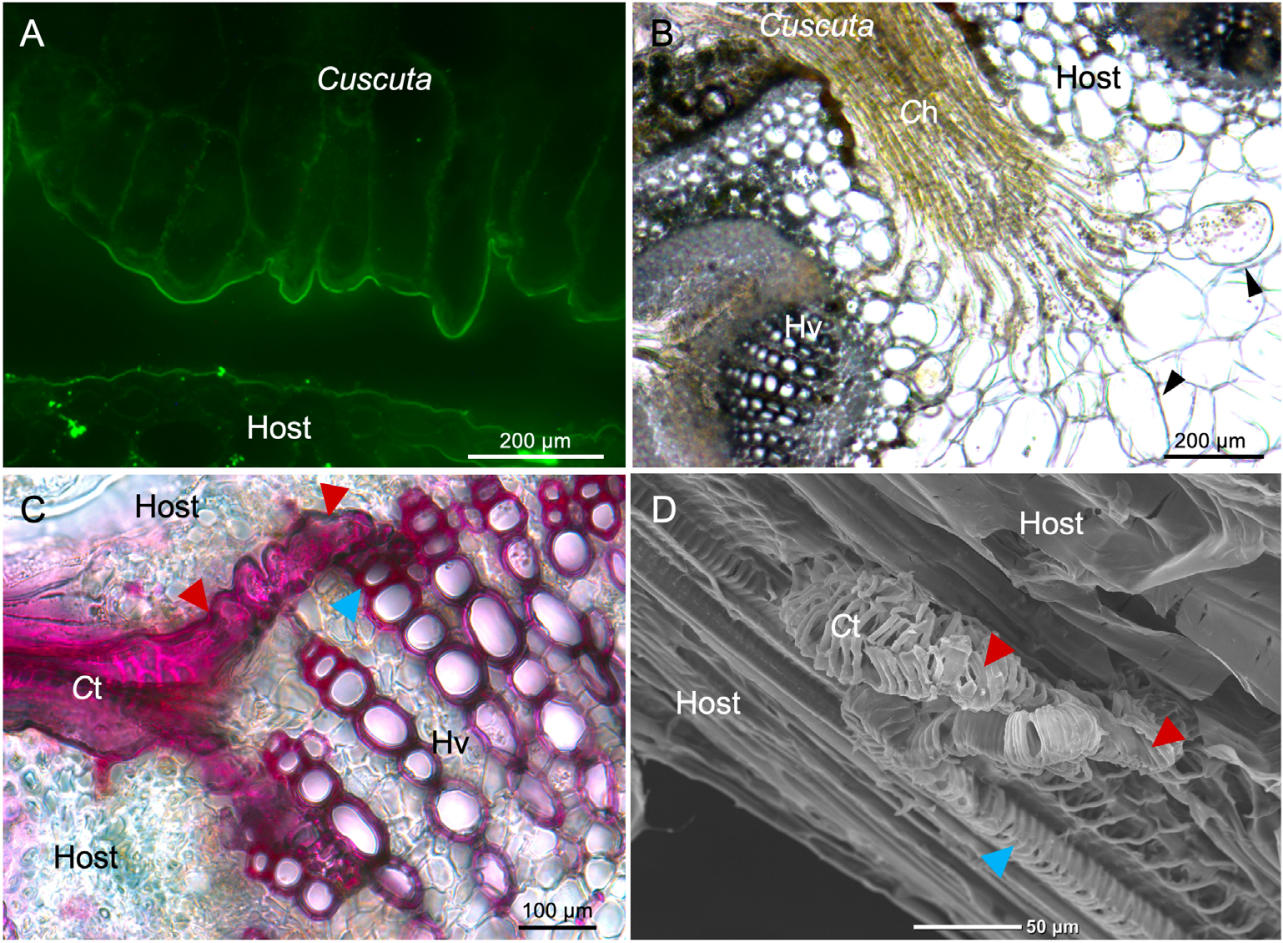
Histological analysis of the parasitic processes of *C. campestris* invading the host plant (*Trifolium pretence*). (A) Immunohistochemical analysis of the contact site between *C. campestris* and host. The *Cuscuta* stem, separated from the host, was cross‐sectioned and stained with an LM6 antibody using the Tyramide Signal Amplification method (Invitrogen‐Molecular Probes). LM6 recognises pectic polysaccharides and AGPs (Willats *et al*. 1998). Photograph was taken using fluorescein isothiocyanate filters fitted to an epifluorescence microscope (DMRXP; Leica). (B) Light micrograph of the haustorium of *C*. *campestris* invading the host stem. Arrowheads indicate search hyphae. Arrowheads indicate search hyphae. *C*h, *Cuscuta* haustorium; Hv, host vasculature. (C) Superficially observed features of the connecting site of the tracheary elements of *Cuscuta* and its host. Cross‐sections of the haustorium penetrating the host tissue were obtained according to the method by Hongo *et al*. (2012); they were stained and clarified with lignin and observed under a stereomicroscope. Red and blue arrowheads indicate *Cuscuta* and host tracheary elements, respectively. *C*t, *Cuscuta* tracheary element; Hv, host vasculature. (D) Stereoscopic observation of the connect site connecting the *Cuscuta* tracheary elements of *Cuscuta* and the host. *Cusucta‐*infected host was cut into cross‐sections to expose the connection site. The cross‐section was immediately freeze‐dried and examined using SEM (JSM‐6610F; JEOL Co. Ltd.). The tracheary element of *Cuscuta* leads from the back to the front of the scene. Red and blue arrowheads indicate *Cuscuta* and host tracheary elements, respectively. *C*t also indicates *Cuscuta* tracheary element.

The *Cuscuta* stem initially performs exaggerated circumnutation to find a host and then switches to a coiling movement upon contact with the host (Yokoyama *et al*. 2023). Sequential tight coil is caused by gelatinous fibres (G‐fibres), which are characterised by a thickened innermost cell wall layer, resulting in close contact between the *Cuscuta* and the host stem. G‐fibres have been found widely in twining vines, including *Ipomea nil*, a close relative of *Cuscuta* species (Bowling & Vaughn 2009). G‐fibres may have commonly evolved in structural parasites using other plants. The epidermal cells of the *Cuscuta* stem secrete adhesive substances at the point of contact with the host, thus cementing *Cuscuta* onto the host (Fig. 2A). Through the formation of this ‘cement layer’ a firm adhesion between *Cuscuta* and the host is achieved, which promotes the invasion of haustoria into the host (Shimizu & Aoki 2019). Homogalacturonan (HG), one of the main components of adhesives, is a cell wall pectic polysaccharide (Vaughn 2003; Hozumi *et al*. 2017). HG is secreted in a highly methyl esterified form but is predominantly demethylesterified in the cement layer, possibly by pectin methylesterases (PME) (Vaughn 2002). Low‐methyl‐esterified HG can form intermolecular Ca^2+^ bonds during gelation (Ridley *et al*. 2001). The mechanical properties of the calcium‐mediated pectin gels play a critical role in cell adhesion to cell junctions (Willats *et al*. 2001; Wolf *et al*. 2009; Bou Daher & Braybrook 2015; Du *et al*. 2022). Pectin gelation is essential for the adhesion of *Cuscuta* spp. to host cells.

Another important component of adhesives is arabinogalactan proteins (AGPs), which belong to a large family of cell wall glycoproteins. Some AGP genes are specifically upregulated during the adhesion phase (Hozumi *et al*. 2017). Additionally, immunolabelling has shown that AGPs accumulate in the epidermal cell walls of *Cuscuta* stems upon contact with the host (Johnsen *et al*. 2015; Striberny & Krause 2015; Hozumi *et al*. 2017). In *C. reflexa*, *attAGP*‐targeted gene silencing indicated a correlation between *attAGP* expression levels and the strength of parasite binding to the host, suggesting a positive contribution of AGPs to parasite–host attachment (Albert *et al*. 2006). High abundance of HG and AGPs have also been reported during the adhesion of the facultative root parasites *Phtheirospermum japonicum* and *Rhinanthus minor*, which may represent a case of convergent evolution during the adhesion of parasitic plants to the host (Pielach *et al*. 2014; Leso *et al*. 2024).

Adhesion was also observed during root fusion, plant grafting, and climbing (Okayasu *et al*. 2021). In particular, pectic polysaccharides and AGPs have also been identified as predominant components of the adhesive substances responsible for the adhesion behaviour of climbing plants, such as English ivy and *Parthenocissus quinquefolia* (Bowling & Vaughn 2008; Huang *et al*. 2016). In the proposed molecular model for the ivy‐derived adhesive, the AGP‐rich nanoparticles in the mucilage exuded from the attachment site promote the generation of strong adhesion, and Ca^2+^‐driven cross‐linking between the carboxyl groups of uronic acid residues within AGPs and pectic polysaccharides causes effective mechanical interlocking at the interface (Huang *et al*. 2016). Although these nanoparticles have not been found in *Cuscuta* spp., plant adhesion may be mediated by a common mechanism at the contact site.

## MUTUAL RECOGNITION THROUGH A WALL

The plant cell wall, which exists on the contact surface of *Cuscuta* species and the host plant, plays a significant role in the mutual recognition of both *Cuscuta* parasite control and host defence responses. The description of the mutual recognition is further divided into two sections: (1) Recognition of the parasite by the host; (2) Recognition of the host by parasites.

### Parasite recognition by the host

Plants have evolved receptors that recognise microbe‐associated molecular patterns (MAMPs), including the cell wall components of bacteria and fungi, and provide immediate defence against microbial infections (Newman *et al*. 2013). However, few studies have reported host plant recognition by the parasites in plant parasitism, with the exception of *Solanum lycopersicum*. This cultivated tomato species recognises *C. reflexa* and shows resistance to parasites because of a hypersensitive response occurring in the early penetration phase (Kaiser *et al*. 2015). *S. lycopersicum* has Cuscuta receptor 1 (CuRe1) as a unique cell surface receptor for the detection of *C. reflexa* (Hegenauer *et al*. 2016). In contrast, *C*. *reflexa* GRP (CrGRP), a binding ligand for CuRe1, acts as a MAMP that specifically triggers a hypersensitivity response in a CuRe1‐dependent manner (Hegenauer *et al*. 2020).

The plant GRP superfamily comprises a large group of proteins that share a high‐glycine region arranged as (Gly)_*n*_‐X repeats. Although no function has been demonstrated for CrGRP in *C. reflexa*, apoplastic GRPs are the major components of the cell wall, including the protoxylem structures (Ryser *et al*. 2004; Yokoyama & Nishitani 2006). Additionally, some GRPs interact with cell wall‐associated kinases (WAKs), which connect the cytoplasm to the extracellular matrix, and are thought to be required for cell wall integrity and stress‐related responses (Park *et al*. 2001; Wang *et al*. 2009; Kohorn & Kohorn 2012). CpGRP1 forms a complex with CpWAK1 and interacts with pectins, depending on the methyl‐esterification status of the pectin in *Craterostigma plantagineum* (Giarola *et al*. 2016). Interactions between CpGRP1, CpWAK1, and pectins have been suggested to play a role in detecting dehydration‐induced cell wall changes, thereby activating dehydration‐induced signalling pathways (Jung *et al*. 2019). While CrGRP is perceived as a molecular pattern for the recognition of *C. reflexa* by *S. lycopersicum*, the complex containing CrGRP may play a role in the perception of cell wall changes by contact with the host in *C. reflexa*.

### Host recognition by the parasites

A recent study showed that *C. campestris* can sense the presence of mannans, a host‐derived CW component, and induce the expression of the corresponding enzymes for haustoria invasion (Bawin *et al*. 2024). However, the role of CW components in inducing haustorial development remains unclear. While lignin‐related compounds exhibit prehaustoria‐inducing activity in root parasites, such as *P. japonicum* and *Striga hermonthica*, no host‐derived CW component causes *Cuscuta* spp to develop haustoria (Cui *et al*. 2018; Furuta *et al*. 2021; Aoki *et al*. 2022). Since stem coiling and haustoria formation can be abiotically induced by tactile stimuli in *Cuscuta* spp., mechanical stimuli by touch appear to be important for attaching to supports and inducing rapid haustoria development in earlier parasite stages (Tada *et al*. 1996; Kaga *et al*. 2020; Bernal‐Galeano *et al*. 2022). After the adhesion stage of the parasite attachment process, host‐derived CW components and host‐produced ethylene were recognised as stimulatory signals for invasion promotion (Narukawa *et al*. 2021; Yokoyama *et al*. 2022; Bawin *et al*. 2024). This process allows *Cuscuta* to strictly distinguish between plant and non‐plant hosts.

## INVASION OF *CUSCUTA* HAUSTORIA

Careful degradation and modification of host CWs indirectly facilitates host invasion by reducing the chances of damage‐associated molecular patterns (DAMPs) being released from host cell walls, which could trigger a host immune response (Fig. 2B) (Du *et al*. 2022). It has been suggested that a cocktail of hydrolytic enzymes is secreted by haustoria to create fissures in host tissue (Nagar *et al*. 1984; Bradley *et al*. 2024). Increased activities of enzymes, such as cellulases, hemicellulases, PMEs and polygalacturonases (PGs) have been found in haustorial and near‐haustorial tissues (Srivastava *et al*. 1994; Singh & Singh 1997; Bar Nun *et al*. 1999; Bar Nun & Mayer 1999; Johnsen & Krause 2014).

The cellulolytic activity of haustoria can be explained by the necessity of the parasite to invade host tissue (Johnsen & Krause 2014). Some types of cellulases have been shown to degrade cellulose, the main component of host cell walls, and possibly hemicelluloses such as xyloglucan and glucomannan (Bawin *et al*. 2024; Edema *et al*. 2024). Interestingly, the B‐type glycoside hydrolase family 9 (GH9) cellulases have been reported to play a role not only in host cell wall degradation during the penetration process of haustoria by *C. campestris* but also in cell–cell adhesion in the grafting healing process with diverse angiosperms by *Nicotiana* species (Notaguchi *et al*. 2020; Edema *et al*. 2024). This suggests that the common underlying mechanisms of B‐type GH9 cellulases play an important role in *Cuscuta*‐host tissue adhesion, vascular connection, and host cell wall degradation during *Cuscuta* invasion.

PMEs and PGs contribute to cell separation at several stages of plant development (Bou Daher & Braybrook 2015). The limited demethylesterification of HG blocks by PME provides substrate‐binding sites for PGs that catalyse the degradation of the main chain of HG (Pelloux *et al*. 2007). Because the HG‐rich middle lamella is a major physical mediator of cell adhesion, HG degradation weakens the connections between cells, leading to cell separation. Similarly, *Cuscuta* PMEs and PGs likely promote host cell separation and facilitate haustorium penetration. PMEs have also been reported to confer pectolytic activity to the infection zone of the root‐parasitic plant *Orobanche cumana* (Losner‐Goshen *et al*. 1998). PME‐regulated HG degradation regulated by PMEs could be a key event in cell separation for haustorium penetration into parasitic plants.

**Fig. 3:**
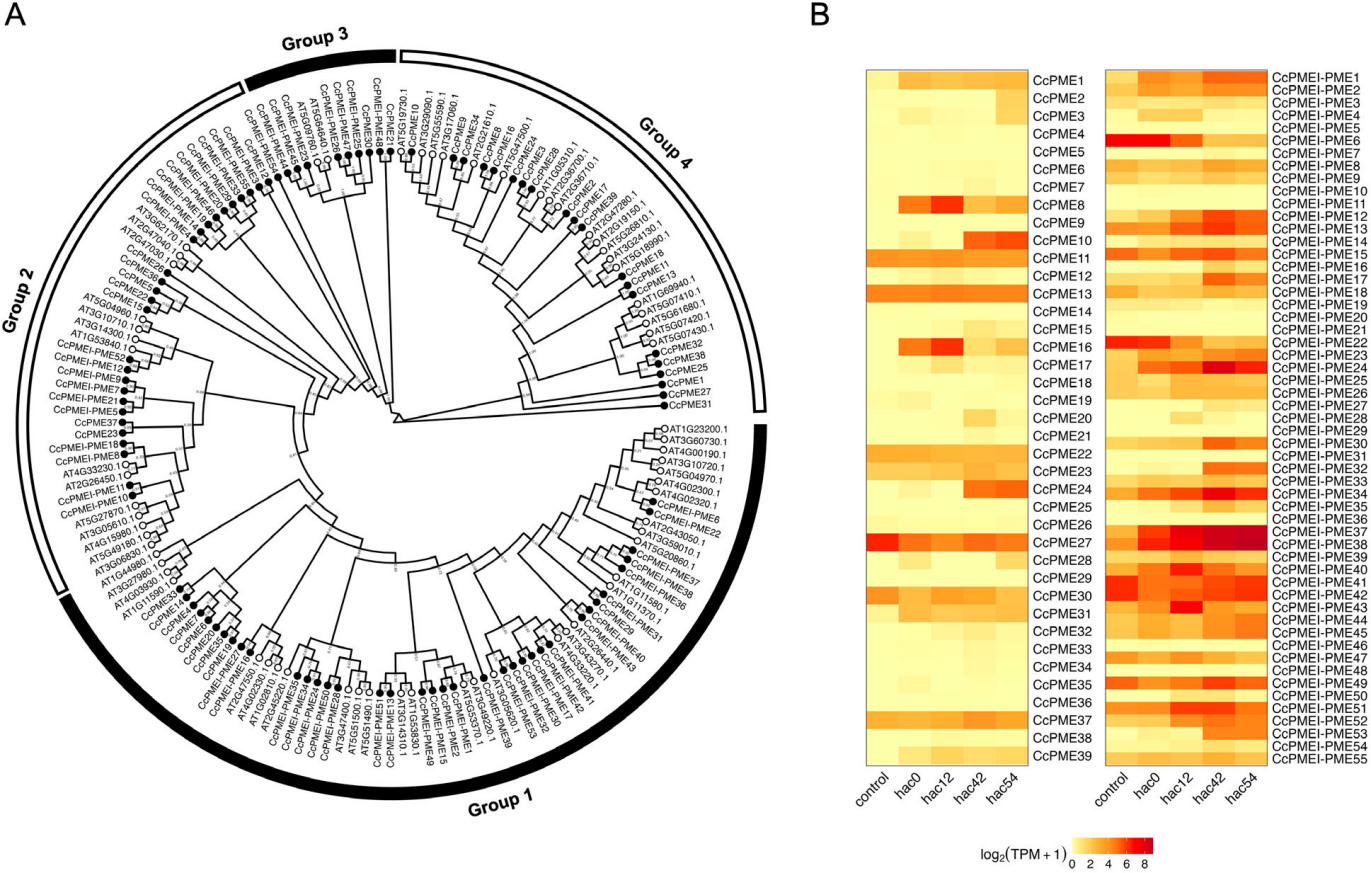
Phylogenetic tree of PME proteins from *C. campestris* and *A. thaliana* and analysis of changes in transcript expression of *CcPMEs*. (A) Phylogenetic tree of the PME proteins of *C. campestris* and *A. thaliana*. Ninety‐four CcPME proteins were identified from the *C. campestris* genome (Cuscuta) available at the National Center for Biotechnology Information (NCBI) and analysed using hmmscan (*E*‐value < 1e–10) in HMMER3 (version 3.4) and the hidden Markov model (HMM) based on the Pfam database (version 37.0) containing the Pectinesterase (PF01095) domain (Eddy 2011; Mistry *et al*. 2021). Following the proposal by Coculo & Lionetti (2022), among the predicted 94 CcPMEs, 55 were ‘CcPMEI‐PME’ (Type‐1) containing both the Pectinesterase and PMEI domains and 39 were ‘CcPME’ (Type‐2) which contained the Pectinesterase domains, but no PMEI domains. The PME subfamilies of PMEs are defined according to Louvet *et al*. (2006). The IDs of the *CcPME* genes are listed in Table S1. The phylogenetic tree was reconstructed using gs2 (version 2.4) which rapidly estimates phylogenetic trees by applying a graph‐splitting method without constructing multiple sequence alignments (Matsui and Iwasaki, 2020). The numbers in the internal nodes indicate the Edge Perturbation (EP) values used to evaluate branch reliabilities. The black circles in the external nodes indicate CcPMEs and the white circles indicate AtPMEs. (B) Analysis of changes in transcript expression of 94 *CcPMEs* during haustorium development based on TPM with RNA‐seq data generated previously by our group (Kaga *et al*. 2020). Hac: Hour(s) after coiling. The colours in the heat map indicate the log‐transformed TPM.

**Fig. 4:**
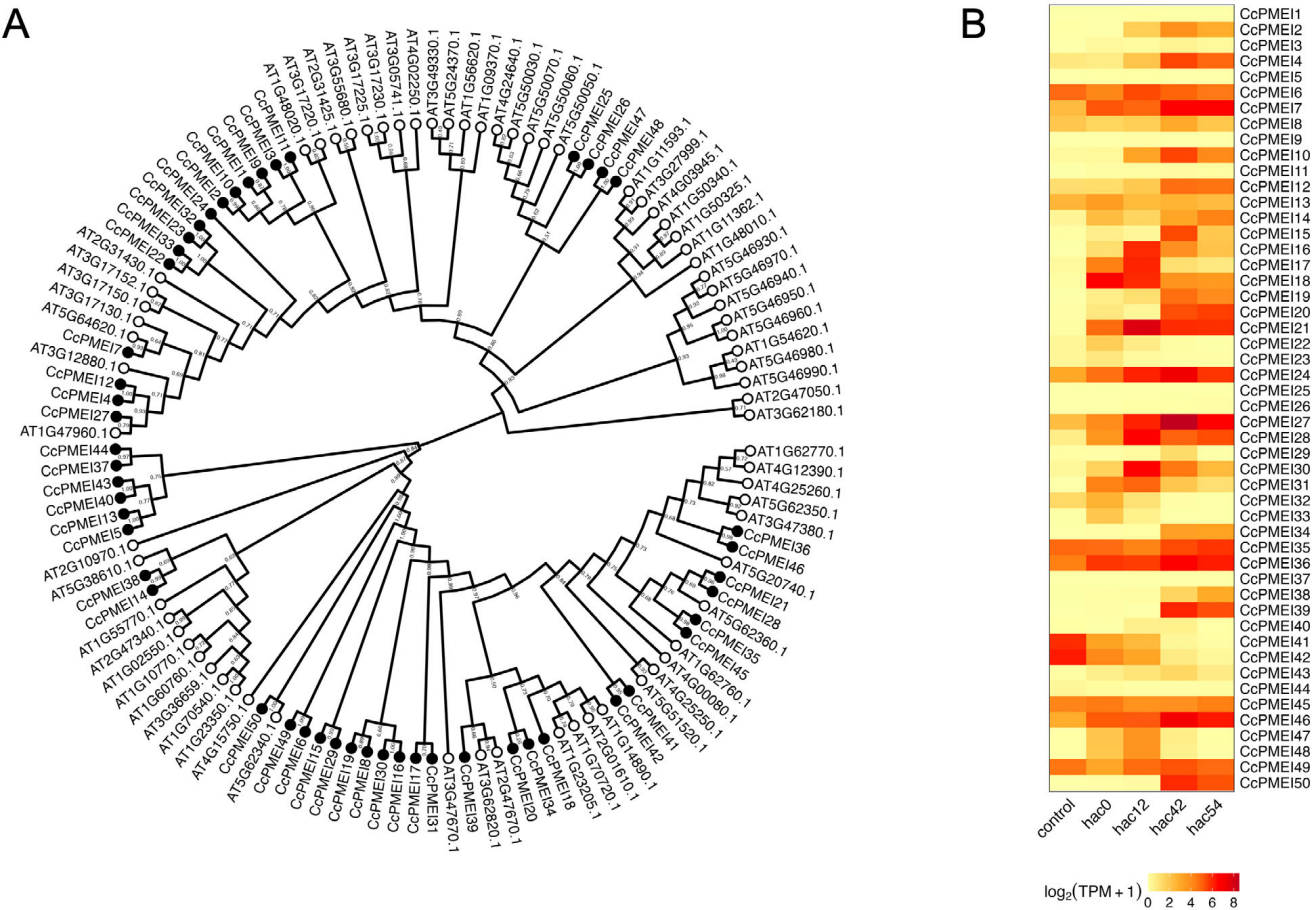
Phylogenetic tree of PMEI proteins from *C. campestris* and *A. thaliana* and analysis of changes in transcript expression of *CcPMEIs*. (A) Phylogenetic tree of PMEI proteins from *C. campestris* and *A. thaliana*. Fifty CcPMEI proteins were identified in the *C. campestris* genome (Cuscuta) using the same method as in Fig. 2 with the PMEI (PF04043) domain. IDs of *CcPMEI* genes are listed in Table xS1. A phylogenetic tree was reconstructed using the method shown in Fig. 2. The numbers in the internal nodes indicate the EP values. The black circles in the external nodes indicate CcPMEIs and the white circles indicate AtPMEIs. (B) Analysis of changes in transcript expression of 50 *CcPMEIs* during haustorium development based on TPM with RNA‐seq data generated previously by our group (Kaga *et al*. 2020). Hac: Hour(s) after coiling. The colours in the heat map indicate the log‐transformed TPM.

The fine‐tuning of HG methyl esterification has been reported to occur through the regulation of PMEs and PME inhibitors (PMEI), which are involved in the post‐transcriptional regulation of PMEs (Jhu *et al*. 2021, 2022). Fifty members of the *CcPMEI* and 94 *CcPME* gene families were identified in the *C. campestris* genome (Table S1). According to the suggestion of Coculo & Lionetti (2022), among the predicted 94 CcPMEs, 55 were ‘CcPMEI‐PME’ (Type‐1) containing both the pectinesterase and PMEI domains and 39 were ‘CcPME’ (Type‐2), which contained the pectinesterase domains, but no PMEI domains. Although their functional differentiation remains to be elucidated, the levels of phylogenetic diversity and abundance of CcPMEIs, CcPMEI‐PMEs and CcPMEs were similar to those in other plant species (Figs. 3A and 4A). As many of these genes showed diverse expression patterns during haustorial invasion, the methyl esterification status of HG in each process may be tightly regulated by the balance between PME activity and post‐translational PME inhibition by PMEIs (Figs. 3B and 4B). For example, it may not be until PME moves away from the haustoria that PMEI represses the PME activity, because *Cuscuta* species need to target host cell walls for degradation without modifying their own cell walls. In this pathway, the PMEI domain of PMEI‐PME may perform a similar function to PMEI (Pelloux *et al*. 2007). A similar example is the cysteine protease called Cuscutain, which has been identified in *C. reflexa* (Bleischwitz *et al*. 2010). Although Cuscutain has a pro‐inhibitory domain attached to an enzymatically active protease domain, it is thought to be cleaved from the enzymatic domain after being secreted from the haustoria. A gradient of the inhibitor domain may occur from the parasite to the host, providing a mechanism for the protein to preferentially modify host tissues over parasite tissues.

In addition to degradation of host walls, changes in the extensibility of host walls may facilitate penetration of haustoria into host tissues. While cell expansion of haustoria is induced by the loosening of their own walls, the softening of the surrounding host walls allows haustoria to pass smoothly through the intercellular spaces in the host tissue. Different levels of pectin methylation were observed in host walls adjacent to the haustoria compared to those not in contact with the haustoria, suggesting that they are involved in host wall extensibility (Johnsen *et al*. 2015; Jhu *et al*. 2021). The elevated activity of xyloglucan endotransglucosylases/hydrolases (XTHs) was also found during the penetration stage of haustoria (Olsen *et al*. 2016). XTHs were originally identified as enzymes that catalysed molecular grafting reactions between xyloglucan molecules (known as XET activity) or the depolymerisation of xyloglucan molecules (known as XEH activity) (Hrmova *et al*. 2022; Ishida & Yokoyama 2022). These two types of XTH genes can be structurally divided into distinct subgroups and both contribute to the extensibility and degradation of CW (Viborg *et al*. 2019). Cell separation processes, such as fruit ripening and organ abscission, involve XEH activity for xyloglucan degradation and XET activity for altering the extensibility of CW (Tsuchiya *et al*. 2015). Both XTH genes are expressed during haustoria penetration in *C. campestris*. However, since the inhibition of XET activity at the Cuscuta–host interface remarkably suppresses the host tissue‐invading capability of haustoria, the XET activity of XTH may be particularly significant for promoting the penetration of haustoria during *Cuscuta* invasion (Olsen & Krause 2017).

## CONNECTION OF TRACHEARY ELEMENTS BETWEEN *CUSCUTA* AND THE HOST

Search hyphae originating from the haustoria transdifferentiated into tracheary elements upon contact with the host xylem and formed a direct connection with the host tracheary element to complete the parasitic linkage (Fig. 2C,D). Similar to the general process of xylem differentiation, trans‐differentiation of search hyphae into tracheary elements is involved in the formation of late‐stage patterned SCWs. The deposition of lignified SCWs may provide mechanical strength and hydrophobicity to the tracheary elements of haustoria, consequently strengthening them to withstand negative pressure by drawing fluids from the host.

SCW cellulose biosynthesis involves cellulose synthase A (CesA), which functions nonredundantly (e.g. CesA4, CesA7 and CesA8 in *Arabidopsis thaliana*) (Taylor *et al*. 2003). In addition, many monolignol biosynthetic enzymes and oxidases are responsible for lignin biosynthesis and polymerisation in SCW (Raes *et al*. 2003; Bonawitz & Chapple 2010). The expression patterns of the orthologues of these genes were closely related to the transdifferentiation of search hyphae into tracheary elements in *C. campestris* (Kaga *et al*. 2020). Additionally, the orthologues of *MYB46* and *MYB83*, which act as regulators of SCW biosynthesis, have similar expression patterns (McCarthy *et al*. 2009; Kaga *et al*. 2020).

The orthologue of *VND7*, which acts upstream of *MYB46* and *MYB83*, is upregulated by contact of search hyphae with the host xylem (Kubo *et al*. 2005; Zhong *et al*. 2007; McCarthy *et al*. 2009; Kaga *et al*. 2020). In contrast, upregulation of the orthologues of master regulators such as MP, LHW, and HB, which act upstream of *VND7*, can be detected in the invading haustoria prior to contact of the search hyphae with the host xylem. These master regulators are involved in the development and proliferation of vascular stem cells (Scarpella *et al*. 2006; Schlereth *et al*. 2010; Ohashi‐Ito *et al*. 2014). Therefore, search hyphae potentially differentiate into vascular stem cells independent of contact with the host xylem. When directly in contact with the host xylem or recognising signalling factors diffused from the host xylem, the search hyphae with vascular stem cell potential may further differentiate into tracheary elements with SCW biosynthesis, possibly through the expression of *CcVND7* (Kaga *et al*. 2020). A unique differentiation system may have evolved to provide a rapid and effective connection between the tracheary elements of *Cuscuta* and its hosts.

## CONCLUDING REMARKS AND FUTURE PERSPECTIVES

Understanding the precise role of CW in multifunctional enzymes is often challenging. For example, the methyl esterification status of HG, which is regulated by PMEs, has completely different biological consequences for different cell types during each parasitic process in *Cuscuta* plants (Fig. 1). PMEs provide mechanical strength to CW for tight attachment during the adhesion process (see section ‘Adhesion between *Cuscuta* and the host plant’), while they induce CW degradation for cell separation at invasion site in host tissue upon haustoria penetration (see section ‘Invasion of *Cuscuta* haustoria’) and increase the CW extensibility of the host cells adjoining haustoria for deeper invasion (Johnsen *et al*. 2015). Both Ca^2+^ cross‐linking and hydrolysis of HG by PME likely enhanced the extensibility of haustoria walls for cell growth. Additionally, PMEs induce HG hydrolysis in search hyphae for tracheary element differentiation and provide mechanical properties to *Cuscuta* cells adjacent to tracheary elements to adjust their external pressure (Nakashima *et al*. 2004; Hongo *et al*. 2012). PMEs and PMEIs, which are involved in the post‐transcriptional regulation of PMEs, are typically encoded by large multigene families, and their family members play different roles in various biological processes. Many of these genes show diverse expression patterns during haustorial invasion, as observed in *C. campestris*. Considering the putative functional diversity and expression profiles, a thorough analysis of the exact roles of individual CW genes in each parasite scenario should provide a basis for a better understanding of the crucial roles of CW dynamics in *Cuscuta* parasitism.

Although *Cuscuta* plants can parasitise various plant species, there is a wide range of compositionally and structurally different CWs between different plant species (Yokoyama 2020). The emphasis here on host CW does not obscure the significant diversity in the wall structure and composition. The molecular system in which *Cuscuta*–host interactions are shared between different types of host CWs is unknown. For example, the haustoria can secrete a cocktail of hydrolytic enzymes that can digest any type of CW, during invasion. Alternatively, *Cuscuta* hydrolytic enzymes may target common components of CW. Similarly, the molecular system involved in the trans‐differentiation of search hyphae into tracheary elements has attracted considerable attention. If host‐derived signalling factors in *Cuscuta* cause search hyphae to transdifferentiate into tracheary elements, they must be ubiquitous molecules involved in the differentiation of tracheary elements in a variety of plant species. Further studies on CW diversity will provide a rich source of information on *Cuscuta*–host interactions.

## AUTHOR CONTRIBUTIONS

MT and RY conceived and wrote the article.

## CONFLICT OF INTEREST

The authors declare no conflicts of interest.

## Supporting information


**Table S1.** Genome‐wide identification of *PME* and *PMEI* gene family members in *C. campestris*.
