# Supplementary figures and images for: Current understanding of the role of the cell wall in Cuscuta parasitism
Source: Plant Biol (Stuttg). 2025 Jun 4;27(7):1235–43. doi: 10.1111/plb.70059 (PMC12631513; doi:10.1111/plb.70059)

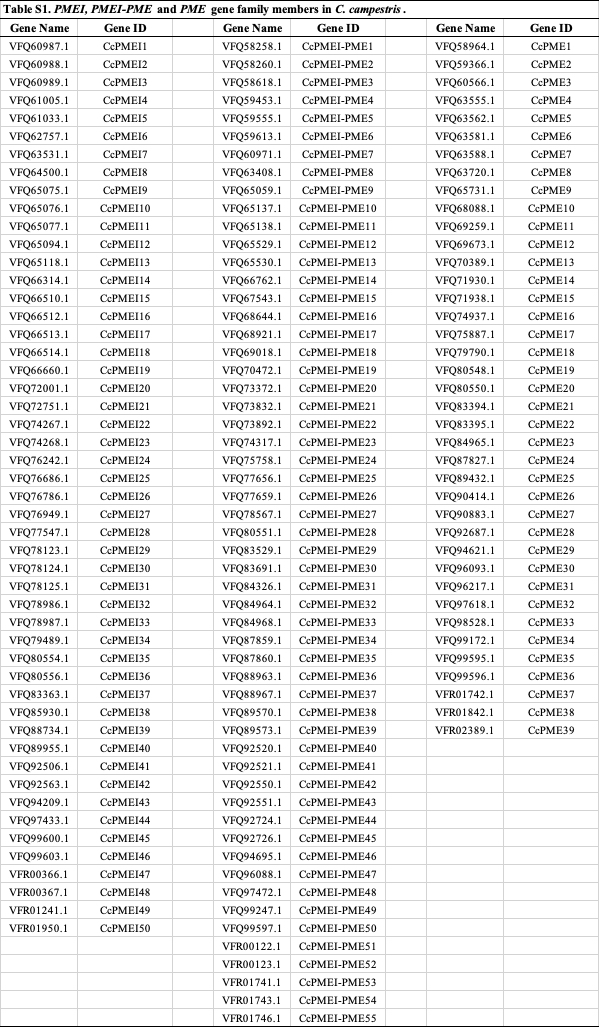

Supplement: Supplementary file 1 — Table S1. Genome‐wide identification of PME and PMEI gene family members in C. campestris. [file PLB-27-1235-s001.jpg]
